# 4T1 Mammary Carcinoma Colonization of Metastatic Niches Is Accelerated by Obesity

**DOI:** 10.3389/fonc.2019.00685

**Published:** 2019-09-20

**Authors:** Gabriela Coeli Menezes Evangelista, Pollyanna Amaral Salvador, Sara Malaguti Andrade Soares, Luciana Rodrigues Carvalho Barros, Felipe Henrique da Cunha Xavier, Luiza Macedo Abdo, Ana Cristina Moura Gualberto, Gilson Costa Macedo, Maria Alejandra Clavijo-Salomon, Jacy Gameiro

**Affiliations:** ^1^Laboratory of Immunology of Infectious and Parasitic Diseases and Obesity, Department of Parasitology, Microbiology, and Immunology, Federal University of Juiz de Fora, Juiz de Fora, Brazil; ^2^Laboratory of Tumor Immunology, Department of Immunology, Institute of Biomedical Sciences, University of São Paulo, São Paulo, Brazil; ^3^Center of Translational Research in Oncology, Institute of Cancer of São Paulo, ICESP, University of São Paulo Medical School, São Paulo, Brazil

**Keywords:** obesity, breast cancer, 4T1 cells, high fat diet, immune-cells infiltration

## Abstract

Breast cancer (BC) remains the leading cause of cancer-related deaths among women, and the chances to develop it are duplicated by obesity. Still, the impact of obesity during BC progression remains less understood. We investigated the role of obesity in tumor progression using the murine model of 4T1 mammary carcinoma in BALB/c female mice, previously high-fat-diet (HFD) fed. HFD induced obesity, metabolic impairment, and high serum and fat leptin levels. After injection of 4T1-cells, HFD-mice accelerated tumor progression and metastasis. 4T1-cells found within HFD-mice metastatic niches presented higher clonogenic potential. 4T1-cells treated *in vitro* with fat-conditioned medium derived from HFD-mice, increased migration capacity through CXCL12 and CCL25 gradients. In HFD-mice, the infiltration and activation of immune cells into tumor-sentinel lymph nodes was overall reduced, except for activated CD4^+^ T cells expressing low CD25 levels. Within the bone marrow, the levels of haematopoiesis-related IL-6 and TNF-α decreased after 4T1-cells injection in HFD-mice whereas increased in the controls, suggesting that upregulation of both cytokines, regardless of the tumor, is disrupted by obesity. Finally, the expression of genes for leptin, CXCR4, and CCR9 (receptors of CXCL12 and CCL25, respectively) was negatively correlated with the infiltration of CD8 T cells in human triple-negative BC tumors from obese patients compared to non-obese. Together, our data present early evidence of systemic networks triggered by obesity that promote BC progression to the metastatic niches. Targeting these pathways might be useful to prevent the rapid BC progression observed among obese patients.

## Introduction

Obesity has become a public health concern worldwide. According to data from the WHO, 13% of the world's adult population was considered obese in 2016 (1). Cancer is the second leading cause of death worldwide, accountable for 8.8 million deaths in 2015 (2). Among all tumors that affect women, 25.2% correspond to breast cancer, which is the world's leading cause of women's death (3). As stated by the International Agency for Research on Cancer—IARC, more than 13 different types of cancer are directly correlated with excess weight or obesity, whereas these conditions are associated with higher risk and lower survival rates in other cancer types (4). Within breast cancer (BC), obesity has been associated with higher morbidity; women who gained between 0.5 and 2 kg/m^2^ after having a breast tumor diagnosed had an elevated risk of death compared with women who maintained their weight (5).

The connection between obesity and breast cancer is, in part given to low-grade chronic systemic inflammation initiated by the accumulation of fat, which attracts inflammatory cells that secrete cytokines, chemokines, and growth factors with pro-tumorigenic potential (6). Though the exact mechanisms that link obesity to breast cancer are not yet fully understood, obesity is known to impair sexual hormones, such as fat tissue-produced oestrogen which is directly linked to the development of breast malignancies (7). Additionally, metabolic changes caused by obesity alter critical hematopoietic and immunologic pathways, such as TNF-α, IL-1, IL-6, and PGE_2_, which also have pro-tumorigenic potential (6, 8).

To better understand obesity, different animal models have been developed (9). Diet-induced obesity is the scenario that most accurately represents the process in humans, as it increases the intake of lipids and/or sugars (such as in western diets), causing energy imbalance between calories consumed and calories burned (10). As a breast cancer model, primary tumor cells taken from spontaneous tumors can rapidly spread to the same areas affected by breast cancer in humans (11). The 4T1 murine breast carcinoma orthotropic model is an ideal system for the studying of the molecular, cellular, and pathological basis of breast cancer and metastatic disease, as well as the studying of therapeutic options.

In 2011, Kim et al. induced obesity in BALB/c female mice through a high-fat diet (HFD) and mammary carcinoma through the injection of 4T1 cells in the mammary glands. It was observed that though there was not much impact on energy intake and body weight, HFD favored tumor growth, angiogenesis, metastases, as well as death among HFD, compared with mice fed with a standard diet (12). However, the systemic mechanisms underlining progression within this model still remain unclear. This work aimed to identify elements by which obesity affects breast cancer progression from primary tumors to sentinel lymph nodes and bone marrow, using an experimental 4T1 murine breast carcinoma model in obese BALB/c female mice.

## Materials and Methods

### Animals

BALB/c female mice aged 4 to 6 weeks were obtained from the colony of the Center for Reproductive Biology, Federal University of Juiz de Fora. This study was performed following the principles of the Basel Declaration and recommendations of the Commission of Ethics in the Use of Animals of the Federal University of Juiz de Fora, approved protocol N°. 038/2014—CEUA.

### Obesity Induction

The animals were randomized before experimentation. Those designated as controls were fed standard commercial food (Nuvilab CR-1™), having 10% of the kilocalories derived from lipids. The animals designated as obese received a high-fat diet, produced in our laboratory, where 57% of the kilocalories were derived from lipids, totalling 35.2% of lipids in the composition. Weight and food intake were monitored weekly for 16 weeks. Food consumption was measured twice a week; values were divided by seven days, and by the number of animals in each group. The Lee index and the weight of retroperitoneal and perigonadal fats were assessed after 16 weeks and 21 days on the high-fat or control diets.

### Biochemical Parameters

A small blood sample from the tail vein of animals, previously fasted for 10 h, was collected for the quantification of fasting glycaemia using a glucometer (ACCUCHECK Performa™). To perform the glucose tolerance test (GTT), 2 g/kg body-weight of a 100 mg/mL glucose solution was administered intraperitoneally, and an additional glycaemia measurement was done after 60 min.

Peripheral blood was collected by cardiac puncture following anesthesia of the animals. Total cholesterol and triglycerides were quantified using commercial kits (Labtest Diagnostica S/A) based on enzymatic colorimetric methods. Readings were performed using the Labmax Progress® (Labtest Diagnostica S/A). Serum leptin and fat leptin concentration were determined by ELISA (R&D Systems™) following the manufacturer recommendations. Optical density reading was performed on a microplate reader (SPECTRAMAX 190, Molecular Devices) at 450 nm. Biochemical parameters were determined on animals after 16 weeks on the high-fat or control diets.

### Cell Culture

Cells of the murine 4T1 mammary carcinoma cell line were kindly given by Dr. Adriana Bonomo. Cells were grown in RPMI-1640 culture medium (GIBCO™) supplemented with 10% fetal bovine serum (SFB—GIBCO™) and 1% antibiotic/antimycotic solution (100 IU/mL penicillin and streptomycin—GIBCO™).

### Orthotopic Breast Cancer Model

A model of transplantable tumors of syngeneic origin was used. After 16 weeks of diet initiation, 5 × 10^4^ 4T1 cells were injected subcutaneously (12), in a total volume of 100 μL, into the region of the first right inguinal mammary gland. All animals continued to be fed their respective diets until euthanasia. Tumor volume was measured using a caliper after 14, 17, 24, and 30 days of induction and calculated according to the formula: largest diameter × (smallest diameter)^2^ × 0.52 (12). After euthanasia, tumors were collected and weighed.

### Metastatic Clonogenic Assay

Tumor-derived peripheral draining inguinal lymph node cells were removed, macerated, and suspended in DMEM medium (GIBCO™) supplemented with 10% FBS. Iliac bones were collected and incubated for 1 h in DMEM medium supplemented with 10% FBS, 0.5 mg/mL collagenase type I (SIGMA ALDRICH™) and 100 μg/mL DNAse (SIGMA-ALDRICH™). Tumor-derived cells and bone marrow cells were harvested and plated at 10^6^ cells/mL per well in 6-well plates, and serial dilutions were performed until the minimum concentration of 10 cells/mL was reached. The culture was maintained at 37°C and 5% CO_2_ for 14 days. Cells were harvested, fixed with methanol (Synth™) and stained with 1% methylene blue (Merck Millipore™) for tumor cells' clusters quantification. The time-points assessed in this assay were selected based on previous work published by Monteiro et al. (13).

### Fat-Conditioned Medium

Perigonadal fats derived from obese and control mice were extracted, weighed, and sterilely washed. Hundred micro gram of fats were manually chopped and cultured with 1 mL of M199 medium (GIBCO™) for 24 h. Culture supernatant was collected, centrifuged to remove cell debris, filtered using a 0.22 μm Millex®GP (Merck Millipore) and stored at −80°C until use.

### 4T1 Cells Migration Assay

Fresh 4T1 cells were pre-cultured for 72 h with 50% fat-conditioned medium (derived from obese or control mice) in RPMI-1640 culture medium (GIBCO™), supplemented with 10% fetal bovine serum and 1% antibiotic and antimycotic (100 IU/mL penicillin and streptomycin—GIBCO™). 10^5^ cells were plated in RPMI-1640 without FBS in the upper chamber of a Transwell® plate (6.5 mm diameter and 8 μm pore size—Corning Incorporated Costar®). As a migratory stimulus, 400 ng/mL of each CXCL12 and CCL25 in RPMI-1640 without FBS was placed in the lower chamber. After 6 h, the membrane of the upper well was fixed and stained with Panotico kit (Laborclin). Cells were counted in a microscope using the NIS-Element BR 3.2 software (Nikon).

### Characterization of Immune Cells by Flow Cytometry

The pools of draining inguinal lymph nodes were harvested and 10^6^ cells per well plated to perform cell labeling with the following antibodies: anti-CD11b (Alexa 488), anti-CD11c (APCCy7), anti-CD80 (APC), and anti-CD86 (PE) for myeloid cells; anti-CD3 (APCCy7), anti-CD8 (Alexa 647), anti-CD4 (FITC), and anti-CD25 (PECy7) for T cells; anti-Foxp3 (PE) for regulatory T cells (all antibodies purchased from BD Biosciences™). The samples were acquired on the BD FACSCanto II™ cytometer and data analyzed using FlowJo v10 (LLC). Gating strategy: Lymphocytes and myeloid cells were initially gated based on size and granularity (FSC X SSC). On the lymphocytes, a gate for CD3^+^ was drawn to label T cells. From this gate, a CD4 vs. CD8 plot was drawn to separate CD4^+^ and CD8^+^ T cells. Within each of these populations, a graph plotting CD25 and FoxP3 was used to delineate regulatory T cells. Cells with larger size and granularity were then selected and gated for CD11b^+^ and CD11c^+^ to select macrophages and DCs, respectively. Within each of these populations, a CD80 vs. CD86 plot was drawn to determine the double positive cells.

### ELISA

The concentration of TNF-α and IL-6 was measured in the culture supernatants of bone marrow cells, previously harvested and plated at 10^7^ cells/mL per well in 96-well culture plate and incubated with 1 ug/mL anti-CD3 (BD Biosciences™) for 72 h. For measurements, commercial kits (R & D Systems) were used following the manufacturer recommendations. Analyses were performed on a microplate reader (SPECTRAMAX 190, Molecular Devices) at 450 nm. Quantification of the cytokines was calculated from the standard curves obtained from the different concentrations of each recombinant protein.

### Bioinformatic Analysis

Microarray data of human triple-negative breast cancer (TNBC) tumors were obtained from GEO, access number GSE76124 (14) and analyzed with limma and Biobase R packages (R version 3.6.0, Bioconductor version 3.9). Women were considered “healthy” or non-obese if BMI ranged from 16 to 24 (*n* = 49), with overweight if BMI ranged from 25 to 29 (*n* = 93), and obese when BMI was >30 (ranged from 30 to 46) (*n* = 56). Heatmap was constructed with pheatmap R package. Spearman correlation tests and plots were made by corrplot R package (15) using immune cells percentage obtained from CIBERSORT (16) output and normalized gene values. The correlation was considered significant if *p*-value < 0.05.

### Statistical Analysis

Graphs and statistical analyses were performed using GraphPad Prism 5.0 (GraphPad Software). After Kolmogorov-Smirnov normality tests, the data were submitted to the unpaired *t*-test or Mann-Whitney test. Data on body weight, food intake, and clonogenic assays were analyzed by two-way ANOVA followed by Sidak's multiple comparisons post-test. Data in graphs are presented as mean values and their corresponding standard deviation. The difference between groups was considered significant when *p* ≤ 0.05.

## Results

### HFD Induces Obesity and Metabolic Impairment

**Figure 1:**
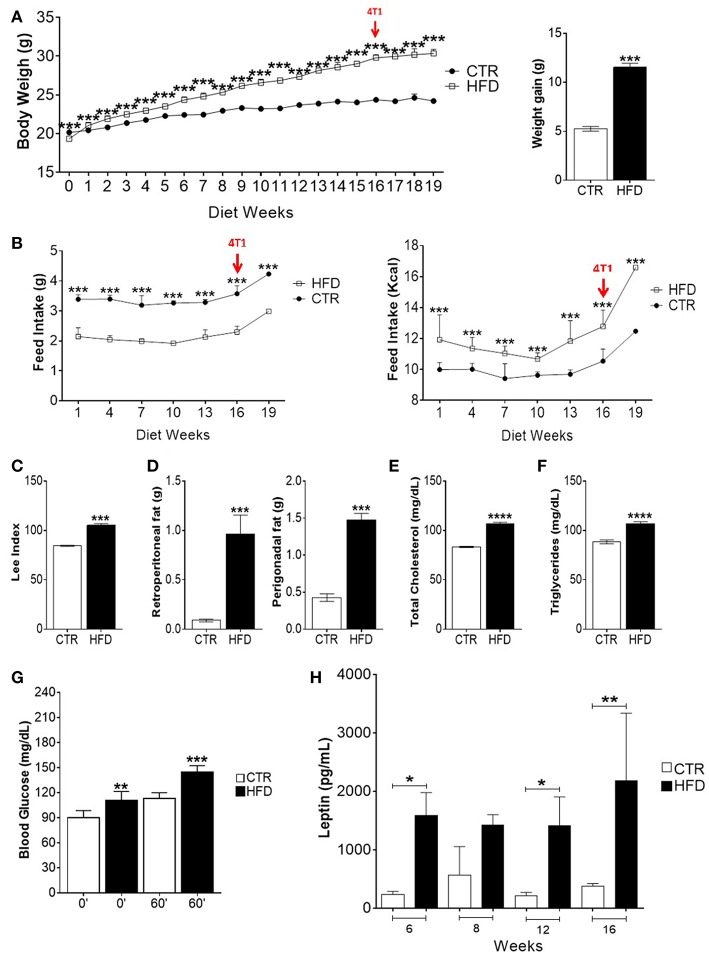
High-fat diet induces obesity, metabolic impairment and high leptin levels in BALB/c mice. **(A)** Body weight gain per week (left) and overall body weight gain (right); **(B)** food intake (left) and caloric intake (right) per week; **(C)** Lee index; **(D)** weight of retroperitoneal and perigonadal fats; **(E)** total serum cholesterol; **(F)** serum triglycerides; **(G)** blood glucose; **(H)** serum leptin. CTR, control animals; HFD, obese animals. Each bar represents the mean ± SD. *Denotes significant differences between obese vs. control group, being *p ≤ 0.05; **p ≤ 0.01; ***p ≤ 0.001; ****p ≤ 0.0001 (*n* = 116 controls and *n* = 116 obese–**A,B**) (*n* = 90 controls and *n* = 75 obese–**C**) (*n* = 112 controls and *n* = 118 obese–**D**) (*n* = 5 controls and *n* = 5 obese–**E–G**).

To evaluate the induction of obesity, corporal, and biochemical changes in control and HFD-fed mice were analyzed. After 2 weeks and through the following 14 weeks, the weight of HFD-fed mice was significantly higher than the weight of control mice; HFD-fed mice were, on average, 4.77 grams heavier than control mice (Figure 1A). During all 16 weeks, HFD-fed mice presented lower mean food intake than control mice, whereas the mean food intake of HFD in kilocalories (Kcal) was higher than mice fed standard diet (Figure 1B). HFD-fed mice presented an increased Lee index (Figure 1C) as well as weightier retroperitoneal and perigonadal fats (Figure 1D). Regarding their metabolic profile, HFD-fed mice presented higher serum levels of total cholesterol (Figure 1E) triglycerides (Figure 1F), glycaemia after receiving glucose (Figure 1G) and leptin (Figure 1H), compared with control mice.

### Tumor Progression Is Accelerated in Obese Mice

**Figure 2:**
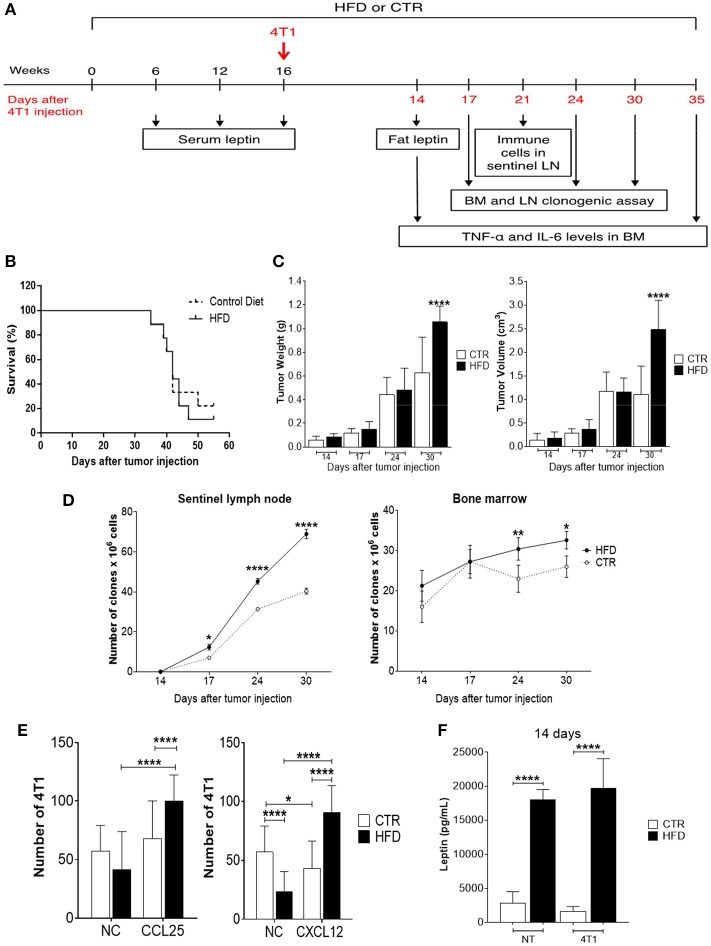
Obese mice exhibit accelerated 4T1 tumor progression and metastasis. **(A)** Experimental timeline; **(B)** survival; **(C)** tumor weight (left) and volume (right); **(D)** number of 4T1 cell clones found in sentinel lymph nodes (left) and bone marrow (right); **(E)** number of fresh 4T1 cells (previously treated with fat-conditioned medium from obese and control animals) found in transwell membranes after migration through CCL25 (left) and CXCL12 (right) gradients; **(F)** leptin levels in fat from obese and control animals with and without 4T1 tumors. CTR, control animals; HFD, obese animals; NC, no chemokines; NT, no tumor. Each bar represents the mean ± SD. *Denotes significant differences between obese vs. control group, being *p ≤ 0.05; **p ≤ 0.01; ***p ≤ 0.001; ****p ≤ 0.0001 (*n* = 5 controls and *n* = 5 obese).

To evaluate the effect of obesity in tumor progression, HFD-fed, and control mice were injected with 4T1 tumor cells after 16 weeks of diet and monitored for several days, during which different experiments were performed (Figure 2A). The survival of obese mice was lower than the survival of control mice, although no statistically significant differences were observed (Figure 2B). As the disease progressed, there was a significant increment in tumor weight and volume of obese mice (Figure 2C). After 55 days of 4T1 cells' injection, 22% of control mice survived compared with 11% within the obese group.

To investigate metastases, a clonogenic assay with 4T1 cells derived from metastatic niches was performed based on previous work done by Monteiro et al. (13). Interestingly, a progressive increase of 4T1 clones was observed in tumor sentinel lymph nodes of obese mice with a significant difference after 17 days of tumor cells injection, compared to controls; similar results were found in the bone marrow which presented an increase of 4T1 cell clones after 24 days of tumor cells injection (Figure 2D).

To explore the mechanism by which 4T1 cells infiltrating the metastatic niches have higher metastatic potential in the obese mice, a transwell migration assay was performed. Fresh 4T1 cells cultured in the presence of conditioned medium containing fat from obese mice exhibited increased migration after CXCL12 and CCL25 stimulus, compared with 4T1 cells cultured with conditioned medium containing fat from control mice (Figure 2E). Notably, increased levels of leptin were found in the conditioned medium containing fat removed from obese mice compared with the conditioned medium containing fat removed from controls (Figure 2F).

### Cancer Sentinel Lymph Nodes of Obese Mice Have Reduced Infiltration and Activation of Immune Cells

**Figure 3:**
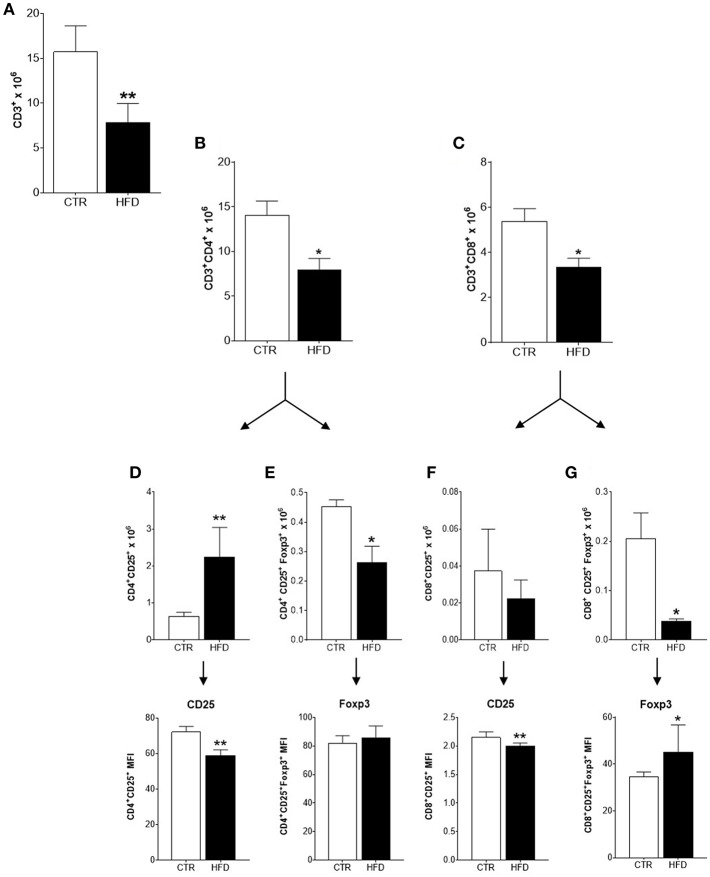
Infiltration and activation of T cells is disrupted in cancer-sentinel lymph nodes of obese mice. **(A)** Number of total CD3^+^ T cells; **(B)** number of total CD3^+^ CD4^+^ T cells; **(C)** number of total CD3^+^ CD8^+^ T cells; **(D)** number of CD4^+^ CD25^+^ T cells (top) and CD25 expression (MFI) within them (bottom); **(E)** number of CD4^+^ CD25^+^ Foxp3^+^ T cells (top) and Foxp3 expression (MFI) within them (bottom); **(F)** number of CD8^+^ CD25^+^ T cells (top) and CD25 expression (MFI) within them (bottom); **(G)** number of CD8^+^ CD25^+^ Foxp3^+^ T cells (top) and Foxp3 expression (MFI) within them (bottom). CTR, control animals; HFD, obese animals. Each bar represents the mean ± SD. *Denotes significant differences between obese vs. control group, being *p ≤ 0.05; **p ≤ 0.01 (*n* = 5 controls and *n* = 5 obese).

To characterize the immune response triggered by the tumor in obese and control mice, the populations of immune cells present in tumor-draining lymph nodes were analyzed. The characterization of immune cells was done on day 21st based on the results obtained from the clonogenic assay: the increment of the clonogenic potential of 4T1 cells was first observed after 17 days of 4T1 injection, reaching higher statistical significance at day 24th. Thus, day 21st was chosen as an average day for the evaluation of immune populations within tumor-draining lymph nodes (Figure 2A). A significant drop in the mean number of total CD3^+^ T cells was observed in obese mice compared with controls (Figure 3A); both, CD4^+^ and CD8^+^ populations were affected (Figures 3B,C). An increased number of activated CD4^+^ CD25^+^ T cells were found in obese mice; however, within this population, CD25 was expressed in lower levels (MFI) than in controls (Figure 3D). The same phenomenon was observed within activated CD8^+^ CD25^+^ T cells (Figure 3F). On the other hand, the number of regulatory CD4^+^ CD25^+^ Foxp3^+^ and CD8^+^ CD25^+^ Foxp3^+^ T cells were significantly decreased in obese mice (Figures 3E,G); however, higher levels (MFI) of the Foxp3 transcription factor was observed within the CD8^+^ regulatory population (Figure 3G). No difference was found on CD25 levels (MFI) within regulatory populations between obese and control mice (data not shown). CD25 levels were higher in regulatory compared to non-regulatory T cells, regardless if they were found in the lymph nodes of obese or control mice (data not shown).

**Figure 4:**
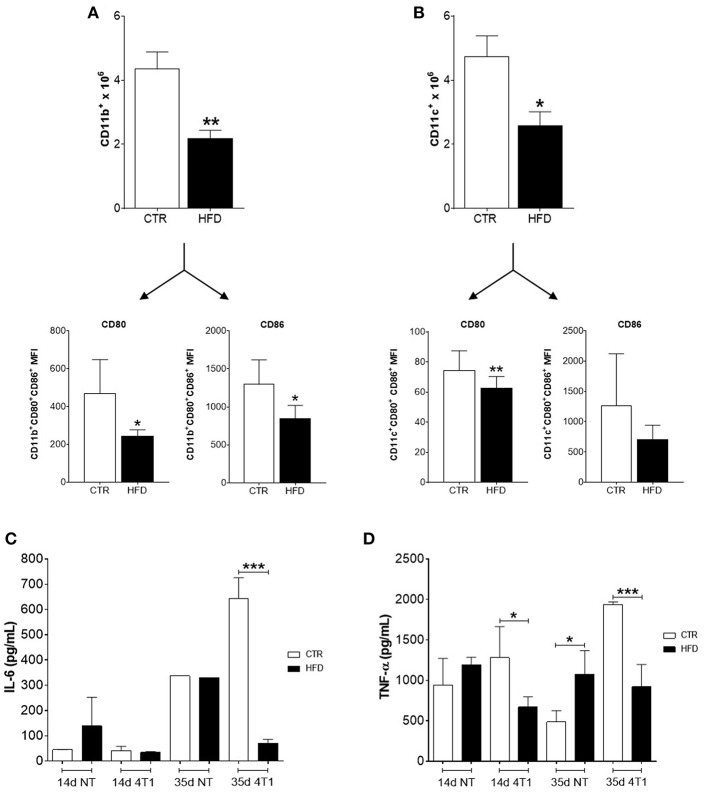
Obese mice exhibit reduced infiltration of myeloid cells within cancer-sentinel lymph nodes and deregulation of haematopoiesis-related cytokines in the bone marrow. **(A)** Number of total CD11b^+^ macrophages (top) and expression (MFI) of CD80 and CD86 within the CD11b^+^ CD80^+^ CD86^+^ population (bottom); **(B)** Number of total CD11c^+^ dendritic cells (top) and expression (MFI) of CD80 and CD86 within the CD11c^+^ CD80^+^ CD86^+^ population (bottom); **(C)** Levels of IL-6 and **(D)** TNF-α in the bone marrow of obese and control animals with and without 4T1 tumors. CTR, control animals; HFD, obese animals; NT, no tumor. Each bar represents the mean ± SD. *Denotes significant differences between obese vs. control group, being *p ≤ 0.05; **p ≤ 0.01; ***p ≤ 0.001 (*n* = 5 controls and *n* = 5 obese).

Regarding myeloid populations, numbers of CD11b^+^ macrophages, and CD11c^+^ dendritic cells (DCs) were also decreased in draining lymph nodes of obese mice compared with controls (Figures 4A,B); both cells also presented low expression (MFI) of co-stimulatory molecules CD80 and CD86.

### Obesity Impairs Levels of Bone Marrow-Derived Cytokines in Mice Bearing 4T1 Tumors

To further investigate the role of obesity in favoring the disruption of haematopoiesis promoted by the increased metastatic potential of 4T1 cells, the level of two cytokines necessary for the development of lymphocyte and myeloid precursors in the bone marrow were analyzed. Notably, only obese mice that received 4T1 cells have both levels of IL-6 (Figure 4C) and TNF-α (Figure 4D) significantly reduced in the bone marrow, compared to controls and obese mice without tumors.

### Reduced Infiltration of CD8 T Cells in Human Triple-Negative BC Tumors From Obese Patients Is Correlated With Leptin, CXCR4, and CCR9

**Figure 5:**
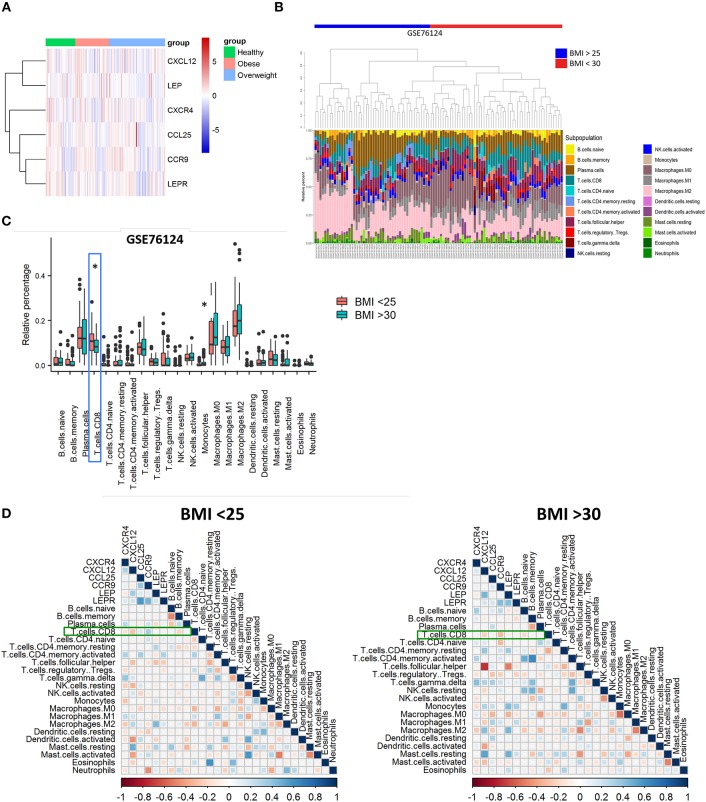
Poor infiltration of CD8 T cells in human triple-negative BC tumors from obese patients is correlated with leptin, CXCR4, and CCR9. **(A)** Gene expression heatmap of genes of interest on primary triple-negative BC tumors from obese and non-obese “healthy” patients. **(B)** Abundance and **(C)** relative percentage of CIBERSORT populations of immune cells infiltrating primary triple-negative BC tumors from obese and non-obese “healthy” patients. **(D)** Correlation between immune cell populations and CXCR4/CXCL12, CCR9/CCL25, and Leptin/Leptin receptor (LEP/LEPR) in primary triple-negative BC tumors from obese and non-obese “healthy” patients. Blue squares represent positive correlations and red squares represent negative correlations where statistically significant differences were found; White dots represent correlations where no statistically significant differences were found. *p ≤ 0.05 (*n* = 49 “healthy” or non-obese patients, *n* = 93 overweighted patients and *n* = 56 obese patients from the GSE76124 dataset).

Based on our murine model, we hypothesized that CXCR4 and CCR9 receptors might be upregulated in breast cancer cells derived from primary tumors of obese individuals. To investigate this, we performed a bioinformatic analysis using microarray data from the GEO GSE76124 dataset. No differences were found in the expression of *CXCL12, LEP, CXCR4, CCL25, CCR9*, or *LEPR* genes in primary triple-negative BC tumors from non-obese (“healthy”), obese or overweighted patients (Figure 5A). Using the CIBERSORT tool, we found that CD8 T cells were significantly decreased in obese patients compared to non-obese (Figures 5B,C). Our correlation analysis (Figure 5D) showed that *CXCL12* and *CXCR4* had a negative correlation with CD8 T cells on both groups. In obese patients (BMI >30), *CCR9* was negatively correlated with CD8 T cell infiltration, whereas was positively correlated, together with *CCL25* in the non-obese (BMI <25). On the other hand, *LEP* was positively correlated with its receptor, *LEPR* only in obese patients. *LEP* and *LEPR* were negatively correlated with CD8 T cell infiltration in the obese, whereas no correlation was found in the non-obese. The positive correlation between *LEP* and *CXCL12* was higher in the obese, whereas the positive correlation between *LEPR* and *CCL25* was higher in the non-obese. Despite being increased in the obese, the LEP-CXCL12-CCR4 network could play a negative role in breast cancer *per se*, whereas obesity might play a role in the functional switch of the LEPR-CCR9-CCL25 network, from promoting tumor immune infiltration in the non-obese, to ultimately favor tumor metastasis in the obese.

## Discussion

**Figure 6:**
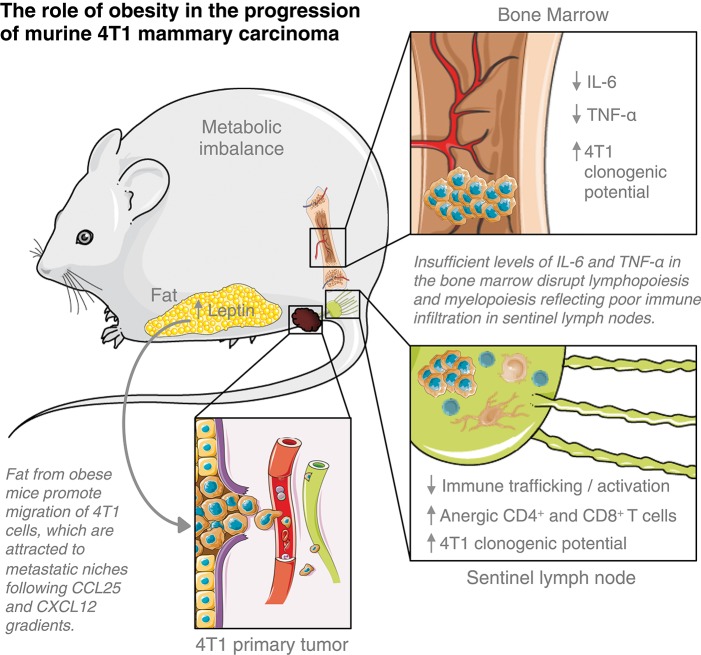
Elements of breast cancer progression triggered by obesity. This figure is a derivative created by the author MC-S using images from Servier Medical Art by Les Laboratoires Servier (https://smart.servier.com/). Original images are licensed under a Creative Commons Attribution 3.0 Unported License (https://creativecommons.org/licenses/by/3.0/).

Obesity is a significant risk factor for the development of breast malignancies. Still, the mechanisms underlining breast cancer progression among obese women remain unclear. Using a diet-induced obesity murine model of 4T1 mammary carcinoma in BALB/c female mice previously fed with a HFD, we showed that fat derived from obese mice favored tumor progression by accelerating the metastatic potential of 4T1 cells within the metastatic niches, thus, disrupting anti-tumoral cell-mediated immunity in tumor-draining lymph nodes and deregulating haematopoiesis-related cytokines in the bone marrow (Figure 6).

Leptin has been acknowledged to be a critical element of the obesity-related progression and malignancy of breast cancer (17). Leptin can directly remodel the tumor microenvironment by inducing metabolic changes in tumor cells and recruiting immune cells such as monocytes, macrophages and myeloid-derived suppressor cells (MDSCs) able to produce proinflammatory cytokines that sustain angiogenesis and tumor growth (18). It was previously shown that the proliferation of 4T1 cells *in vitro* increased by the addition of leptin; also, that the inhibition of leptin-mediated signaling on these cells reduces its proliferation and the growth of 4T1 tumors *in vivo* (19). We showed that fat derived from obese mice, which contain high levels of leptin, induces the migration of 4T1 cells through a *transwell* membrane, following the gradient of CCL25 and CXCL12; although both chemokines recruit immune cells to inflammatory tissues, they have also been linked to invasion and metastasis of cancer cells (20, 21). These findings are in agreement with the increased clonogenic potential of 4T1 cells found in tumor-draining lymph nodes and bone marrow. Leptin has been found to increase the levels of CXCR4 (the CXCL12 receptor) in bone marrow-derived mesenchymal stem cells (22) and to increased CCR9 expression (the CCL25 receptor) on hepatic stellate cells (23). The CXCL12/CXCR4 axis is strongly correlated with breast cancer aggressiveness and metastasis (20), whereas the CCL25/CCR9 axis provides chemotherapy resistance to breast cancer cells (21). Interestingly, from the analysis of the microarray data of the GEO GSE76124 dataset, we observed that the genes for leptin, CXCR4, and CCR9 are correlated with reduced infiltration of CD8 T cells in human triple-negative BC tumors from obese patients. CD8 T cells are considered a favorable prognosis marker for several cancer types, including breast cancer (24, 25); worse outcomes are expected in patients with tumors poorly infiltrated by them, suggesting that obese patients might have disease progression sooner than non-obese, as we observed in our 4T1 mice model. Our data provide a plausible explanation by which obesity worsen breast cancer progression through expose breast cancer cells to high leptin levels, that ultimately favor the chemokine networks that, not only hamper CD8 T cell infiltration to the tumor but instead, are used by cancer cells to spread and metastasize.

The immune profiling of tumor-sentinel lymph nodes is a clinically valuable tool to predict breast cancer prognosis (26). We found that immune cells poorly infiltrate tumor-draining lymph nodes of obese mice; all populations were decreased, including subsets related to tumor progression, such as regulatory T cells and macrophages. Interestingly, an increased number of CD4^+^ CD25^+^ T cells were found in obese mice, expressing lower levels of CD25 (the IL-2R alpha chain) than CD4^+^ CD25^+^ T cells found in non-obese mice. Downregulation of CD25 within activated T cells is linked to defective signaling that leads to unresponsive or anergic T cell clones (27, 28). This phenomenon might be supported by the reduced expression of co-stimulatory molecules found in DCs and macrophages within tumor-draining lymph nodes. Also, CD25 lost within activated T cells might be induced by competition for IL-2 between activated and regulatory T cells expressing higher levels of Foxp3, which has been directly associated with their potential to suppress the activation of effector T cells and correlated with cancer progression (29, 30).

Typically, myeloid cells are increased within the tumor microenvironment and pre-metastatic niches to promote tumor survival and metastases (31, 32). Obesity seems to disturb this scenario; after 6 weeks of HFD, bone marrow malfunction is induced by a mechanism depending on LPS originated from bacteria (translocated from the gut to the bloodstream) that stimulate, via TLR4, myelopoiesis instead of lymphopoiesis (33). However, as obesity accelerates tumor progression, the increment of DCs or macrophages is not reflected within tumor-draining lymph nodes, and both populations within them were also compromised. A possible boost of MDSCs cannot be ruled out; although it was not directly measured in our model, MDSCs are induced after prolonged chronic inflammation, such as obesity, once their development can be triggered by fatty acids (34, 35). Within the metastatic niches, these cells have been shown to interfere and suppress tumor-specific T cell responses as well as myeloid cells (36) whereas their blockage can restore DCs and macrophages activation (37). Instead of a consequence of the high-fat diet *per se* (38), immunosuppression could result as a consequence of the accelerated progression promoted by the HFD, which could also cause trafficking disruption of immune cells to the lymph nodes. Indeed, cancer-induced remodeling of high endothelial venules (HEVs) is a distinctive feature of pre-metastatic sentinel lymph nodes, which results in a functional shifting of HEVs that facilitates the blood flow into the tumors (39). Also, immunosuppression in obese animals may be linked to a possible hematopoietic dysfunction caused by bone marrow metastases. A reduction in blood T cell populations of mice HFD-fed has been attributed to the loss of balance between skeletal and immune systems, resulting in the dysfunction of haematopoiesis (40). Within the cytokines involved in the process, IL-6 and TNF-α decreased in the bone marrow of obese animals, whereas levels of both cytokines increased in the non-obese, after 4T1 cells injection. Increased pro-inflammatory cytokines such as TNF-α, IL-1, IL-6, and PGE_2_ are hallmarks of obesity and obesity-induced breast cancer development (8, 41). However, in a state of accelerated progression induced by obesity, a detriment of pro-inflammatory IL-6 and TNF-α production within the bone marrow may support the disruption of both myelopoiesis and lymphopoiesis (31, 42, 43). Low levels of both cytokines could also be caused by its consumption by 4T1 cells infiltrating the bone marrow, which are known to express IL-6 and TNF-α receptors and to trigger their signaling pathways after *in vitro* treatment with the cytokines (44).

In conclusion, our work present early evidence by which obesity promotes breast cancer progression from a systemic point of view, beyond the tumor microenvironment. Moreover, we show that the changes observed within the tumor (12) do not reflect on the systemic response here assessed. Still, more studies are needed to connect the leptin-CXCL12-CCL25 axis and to define the possible role of MDSCs in the breast cancer accelerated immunosuppression and progression promoted by high-fat-diet-induced obesity. Targeting these pathways, probably involved in the paving of pre-metastatic niches, might be useful to prevent the rapid breast cancer progression observed among obese patients.

## Data Availability

All datasets generated for this study are included in the manuscript/supplementary files.

## Ethics Statement

This study was carried out in accordance with the principles of the Basel Declaration and recommendations of Commission of Ethics in the Use of Animals of the Federal University of Juiz de Fora, that approval the protocol N°. 038/2014–CEUA.

## Author Contributions

GE, PS, and JG conceived the experiments. GE, PS, SS, FX, LA, AG, and GM conducted the experiments. GE and PS analyzed the results. MC-S and LB performed the bioinformatic analysis. MC-S interpreted the data and wrote the manuscript along with GE and PS. All authors reviewed the final version of the manuscript.

### Conflict of Interest Statement

The authors declare that the research was conducted in the absence of any commercial or financial relationships that could be construed as a potential conflict of interest.
